# Evaluation of calcium cyanamide addition during co-composting of manure and maize straw in a forced-aeration static-pile system

**DOI:** 10.1186/s40201-016-0258-7

**Published:** 2016-10-26

**Authors:** Huasai Simujide, Chen Aorigele, Chun-Jie Wang, Tian-Hua Zhang, Bai Manda

**Affiliations:** 1 College of Animal Science, Inner Mongolia Agricultural University, Zhaowuda road, 306, 010018 Hohhot, China; 2 College of Veterinary Medicine, Inner Mongolia Agricultural University, Zhaowuda road, 306, 010018 Hohhot, China

**Keywords:** Calcium cyanamide, Antimicrobial agent, Manure, Composting, Pathogen

## Abstract

**Background:**

Composting is one of the most environmentally friendly treatments to inactivate pathogenic organisms or reduce them to acceptable levels. However, even under thermal conditions, some pathogenic organisms such as *E. coli* could exist for a long time in composting. Such great persistence may increase the possibility of outbreaks of these organisms and further increase the environmental load. Calcium cyanamide (CaCN_2_) has recently been recognized to have the fungicidal effect on the pathogens of the soilborne diseases. So, the present study determined the effect of CaCN_2_ addition on composting progress as an antimicrobial agent and an amendment during forced-aeration static-pile composting of cow manure, which was mainly aimed to inhibit the pathogens that had not been inactivated by heat during composting.

**Methods:**

The mixtures of dairy cow manure and maize straw with addition of 2 % CaCN_2_ or no addition were composted for 63 days. The physical, chemical and biological changes in compost mixtures were examined during composting. The data were statistically analyzed using ANOVA procedure from SAS software (version 9.0).

**Results:**

The results showed that the addition of CaCN_2_ significantly increased the maximum temperature and lengthened the duration of the thermophilic phase, and increased the percent T-N but decreased C/N ratio. For microbiological test, the addition of CaCN_2_ shortened the time to inactivate *E. coli*, and increased the total average population of thermophilic bacteria but did not significantly influence that of mesophilic bacteria.

**Conclusion:**

The results indicated that the addition of CaCN_2_, at least at the additive content of 2 % could benefit the thermophilic phase and the composting could quickly reach the sanitary standard during the composting of manure with maize straw in a forced-aeration static-pile system. This finding will contribute to solve the feces disposal problems.

## Background

Cattle manure, a valuable resource because of its nutrient and organic matter contents, is used as a soil fertilizer and represents a low-cost alternative to mineral fertilizers [1]. However, if the appropriate treatment methods are not carried out, manures would become solid wastes rather than valued resources. Serious environmental problems, such as an excessive input of potentially harmful trace metals, inorganic salts and animal pathogens would develop because of inappropriate disposal practices [2, 3].

Composting is not only the most efficient process to produce an agronomically advantageous soil organic amendment, but also one of the most environmentally friendly treatments to inactivate pathogenic organisms or reduce them to acceptable levels [4, 5]. Minimally managed composting processes can reduce *E. coli* and other pathogens in bovine manure [6, 7]. But the survival of the pathogens is greatly different in different systems, for example, in some composting systems even under thermal conditions, some strains of pathogens could exist for a long time or recover after some days. Such persistence increases the likelihood of disease outbreak and further increases the environmental load. Therefore, it is necessary to produce more reliable and realistic methods feasible in both sanitation and recycling of manure.

Calcium cyanamide (CaCN_2_) has mostly been used as a nitrogen fertilizer for a long time. However, some studies have found its fungicidal effect on the pathogens of the soilborne diseases. The CaCN_2_ effectively suppressed *Fusarium solani* f.sp. *cucurbitae* in greenhouse cucumber [8], and was also fungicidal to *Fusarium oxysporum* f.sp. *Cucumberinum* [9]. However, very little is known about its effect on zoonotic microorganisms. Our previous study showed that manure composting would quickly reach the sanitary standard and the quality of the composting products would be improved with the addition of CaCN_2_ during mesophilic composting at laboratory scale [10]. On the basis of previous work, the present research further studied the effect of CaCN_2_ addition on minimally managed composting progress during co-composting of manure and maize straw in a forced-aeration static-pile system. The evaluation of CaCN_2_ effect in different composting system will allow us to broaden our knowledge about its use in composting. Therefore, the two major objectives of this study were (a) to assess the antimicrobial effect of CaCN_2_ during the minimally composting of cattle manure with maize straw and (b) to evaluate its effect on composting process as an amendment during the minimally composting of cattle manure with maize straw.

## Methods

### Experimental design

Composting trial was conducted on a cow farm of Inner Mongolia Autonomous Region of China in autumn (from September to mid-November). Fresh manure of dairy cows which were apparently healthy but had been confirmed to carry pathogenic *E. coli* with serogroups of O1, O6, O8, O9, O78, O98 and O149 in their gastrointestinal tracts was collected and mixed with maize straw (bulking agent). Composting was conducted on a concrete apron, subdivided into two separate compartments, each with floor dimensions 120 × 120 cm. Compartments were isolated from each other by 120 cm high walls, and were all unroofed. Perforated polyvinylchloride (PVC) pipes segregated from the compost piles by closely spaced parallel boards were used at the bottom of compartments to supply adequate oxygen (supplied by a blower), and to maintain uniform mixing. The air supply was provided from the 4th day of the composting. The air-flow rate was 0.5 m^3^ · min^−1^ during the first 2 weeks and then became 0.25 m^3^ · min^−1^. Maize straw was laid 5 cm thick over the boards in order to distribute air equally. And to ensure that all areas of the compostable material were exposed to the required temperature, each pile was covered with a plastic film. Compost piles contained about 500 kg compostable mixtures per compartment at the beginning of each experiment, and were maintained in a roughly conical shape during composting. The piles were classified into test pile and control pile. Solid CaCN_2_ was thoroughly mixed with the test pile at the mixing rate of 2 % by weight, while was not added to the control pile. The mixing rate was based on the previous study, which had showed that manure composting process would not be influenced by the addition of both 2 and 3 % CaCN_2_ during mesophilic composting at laboratory scale [10]. Compost samples in duplicate were collected from each pile by using quartering process at days 0, 1, 3, 4, 9, 14, 21, 28, 35, 42, 49, 56, and 63 for the analysis of different parameters.

### Physico-chemical analysis

Ambient temperature around the compost bins and temperatures within each pile was measured daily at 9:00 AM and 15:00 PM. Daily temperature of the pile was the average temperature of the top, middle and bottom layer in the two measurements. The moisture contents of the samples were determined after oven drying at 105 °C to a constant weight [11]. The pH was determined by a Mettler-Toledo EL20 pH-meter (Mettler-Toledo international trading (Shanghai) Co., Ltd.). Total nitrogen (T-N) and total carbon (T-C) was measured by kjeldahl method and K_2_Cr_2_O_7_ volumetric method [11]. Total phosphorus (T-P) was recovered by sulfuric acid-hydrogen peroxide digestion according to Chinese national standard NY/T 298-1995.

### Seed germination test

Seed germination test was carried out on filter paper lined in petri dishes [12]. Aqueous extract from the composting samples was prepared by shaking for 1 h at a solid:H_2_O ratio of 1:8 (*w/v*, dry weight basis). 5.0 mL each extract was pipetted into a petri dish, and 10 seeds of Chinese cabbage (*Brassica campestris* ssp. *pekinensis*) were evenly placed on the filter paper and incubated at 25 °C for 48 h in darkness. H_2_O was used as an extract of the controls. Each sample was analyzed in quintuplicate. A formula for calculating germination index (GI) was as follows [13]:

$$ GI\left(\%\right)=\kern0.5em \frac{\mathrm{seed}\ \mathrm{germination}\times \mathrm{root}\ \mathrm{length}\ \mathrm{of}\ \mathrm{test}\ \mathrm{pile}}{\mathrm{seed}\ \mathrm{germination}\times \mathrm{root}\ \mathrm{length}\ \mathrm{of}\ \mathrm{control}\ \mathrm{pile}}\times 100\% $$

### Microbiological analysis

Plate counting was performed to determine the populations of fecal indicator bacteria *E. coli*, thermophilic (TB) and mesophilic bacteria (MB) within compost piles. *E. coli* was investigated from eosin-methylene blue agar (EMB) plates and confirmed by Indole Test [14]. Mesophilic and thermophilic bacteria were enumerated on nutrient agar after incubating for 24 h at 30 and 50 °C, respectively.

## Results and discussion

### Physical and chemical analyses

**Fig. 1:**
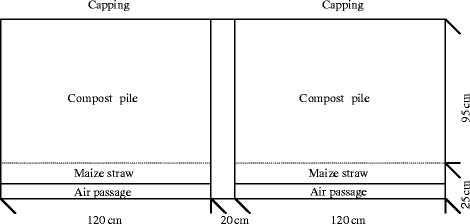
Schematic diagram of the compost pile

Using the aerobic thermophilic composting method, operating conditions of 50–55 °C or greater for 5 to 7 days are recommended by the national sanitary standard [15]. In this trial, the test pile reached temperatures >55 °C for 19 days and >50 °C for 23 days while the control pile recorded lower temperatures which were >50 °C for 9 days when the maximum temperature was only 55 °C at 23 days (Fig. 1). The maximum temperature of 65.5 °C was reached at 29 days for the test pile.

**Fig. 2:**
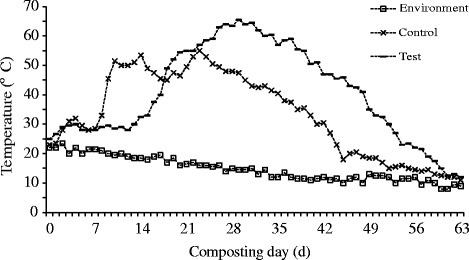
The temperature profile during composting

Some studies suggested the temperatures of 55 to 60 °C for 7 to 14 days as the ideal temperatures for effective composting [16–18]. However, others reported that lower temperatures were still efficient to inactivate bacterial populations such as *E. coli*, the temperatures included 45 °C for 72 h in a study conducted by Lung et al. [19] and 33.5 to 41.5 °C for 7 days by Larney et al. [20]. In our previous study [10], pathogenic *E. coli* with different serotypes were all effectively eliminated during mesophilic composting (peak temperatures 39.0–41.1 °C). On the other hand, the changes in composting temperature during composting are influenced by some factors including compostable materials and composting method. Changa et al. [21] found that the composting temperature reached 60 °C within 10 days during composting of cattle manure blended with a mixture of sawdust and wood shavings, while it was still be around 30 °C for 10 week during composting of the same dairy manure blended with wheat straw and reached a higher level after 6 weeks for hog manure composting. Fuentes et al. [22] reported that the composting temperature rose to about 40 °C within the fist 20 days and kept 43–51 °C at next coming 20 days, and then decreased rapidly and maintained 23 ± 2 °C to the end during aerobic degradation of dairy cattle dung in laboratory-scale reactors for 105 days. In the current study, as shown in Fig. 2, the peak temperatures were achieved at 23 days in the control pile and 29 days in the test pile, indicating that the time to reach the high temperatures was delayed with the addition of CaCN_2_ which was in accordance with our previous laboratory-scale composting studies, and it is always associated with reduced porosity of the composting piles in the beginning. However, the maximum temperature in the test pile was much higher than the control pile, and the duration of high temperatures was also much longer in the former.

The moisture contents of all piles presented the same changes that followed a declining trend. The mean moisture content of the test pile decreased from an initial value of 70.97 to 60.71 %. In the control pile, it decreased from 75.28 to 58.86 %. Water availability is a critical determinant of microbial activity during composting [23]. Several studies reported that the moisture content in fed-batch composting of household biowaste was 30–40 %, at which the microorganisms showed the highest protease activity [24, 25]. Nelson et al. [26] suggested a possible threshold between 50 and 60 % moisture content during cattle manure composting, which has a potential to achieve the best temperature profile and save the energy required to turn the windrows. Many other observations also recommended 50–60 % as the preferred moisture content range during composting [27–29]. Klasse [30] pointed out that the moisture content was one of the important determinants of the decomposition efficiency of CaCN_2_ in soil. Our previous study showed that the elimination of pathogenic *E. coli* by CaCN_2_ during manure composting was most effective in 85 % moisture content condition and was followed by in 65 %. In 45 % moisture content, pathogenic *E. coli* could not be thoroughly inactivated. Based on such result, we aimed to adjust the moisture content of the test pile to 70 % to guarantee both a smooth process for composting and better development for inhibitory effect of CaCN_2_ on *E. coli*.

**Fig. 3:**
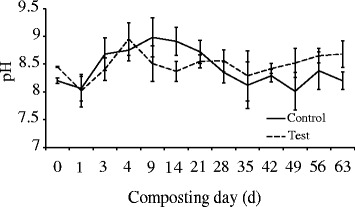
The pH profile during composting

Due to the decomposition of organic matter and production of organic and inorganic acids by the activities of microorganisms, the pH usually decreases at the beginning of composting process, followed by a significant increase with the temperature increases and then decreases during the late stage [31]. The changes in pH in this study showed a similar trend (Fig. 3). A slight decrease in pH occurred at the beginning of composting. This can be explained by a lack of oxygen that may occur at the beginning of composting which would result in the production of acids [32]. The pH 8-9 is known to be a crucial factor during successful composting and in matured compost [33]. Low pH acts as an inhibiting factor for microbial activity in composting process [34, 35]. In this test, the pH fluctuated from 8.02 to 8.95 in the test pile and from 8.01 to 8.98 in the control test during the process (Fig. 3). This implied that both of the two piles were in good physical state and able to facilitate composting [36].

**Fig. 4:**
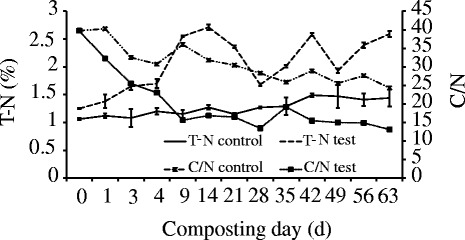
The T-N and C/N profile during composting

Trends in percent T-C for the two piles revealed significant losses in T-C during composting, which was resulted from the constant decomposition of carbon-containing organic substances in compost piles. Percent T-C for the test pile decreased from an initial mean value of 49.66 to 33.90 % at the end of composting representing a loss of 31.74 % of the initial level, when it was from 42.12 to 35.00 % in the control pile which represented a loss of 16.90 % of the initial value. The changes in percent T-N generally showed an increasing trend. The percent T-N in the test pile increased from an initial value of 1.25 to 2.59 % at the end (Fig. 4). The mean values of total percent T-N in the control pile were significantly lower than the test pile and increased from an initial value of 1.06 to 1.44 % at the end of composting. Compostable materials mixed to provide a C/N ranged from 25 to 30 are considered ideal for organic compounds degradation. However, larger ranges have been reported to be acceptable in some studies [37, 38]. Michel et al. [39] suggested that the initial C/N ratio should be greater than 40 during the composting of dairy manure amended with sawdust or straw in order to effectively reduce the N loss. In the present study, the initial values of C/N ratio were 39.73 in the test pile and 39.73 in the control pile (Fig. 4). Then, the C/N ratio in both piles showed a significant declining trend. During composting, the relative concentration of T-N always increases due to the constant loss of organic matter. Therefore, C/N ratio also constantly declines. At the end of composting, the mean value of C/N ratio was 13.09 for the test pile which was significantly lower than that of 24.31 in the control pile.

**Fig. 5:**
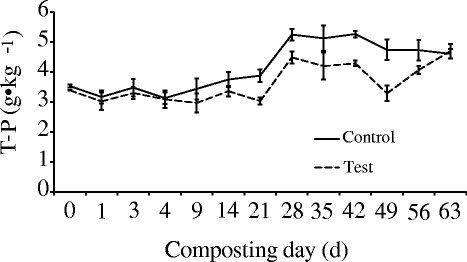
The T-P profile during composting

The changes in T-P of each pile followed the same trend with a subtle decline in the early days, and then an obvious recovery and increase until the end of the experiment (Fig. 5). This indicates that the decomposition of P primarily occurred in the mesophilic phase. The finding is in accordance with the results of Zhang and He [40]. Parkinson et al. [41] reported that the T-P concentrations were elevated whether in turned or static manure composting at the end of all experiments conducted for a minimum of four months. In the present study, the mean T-P content of the test pile increased from an initial value of 3.39 g•kg^−1^ to 4.70 g•kg^−1^ after the whole period of composting and the increasing rate was 38.64 %. In the control pile, it increased from 3.53 g•kg^−1^ to 4.61 g•kg^−1^ with the increasing rate of 30.59 %. The increasing change in T-P is caused by dry matter loss and the gradational content of manure in each pile was responsible for the T-P grads throughout the composting [40, 41].

### Phytotoxicity assay

**Fig. 6:**
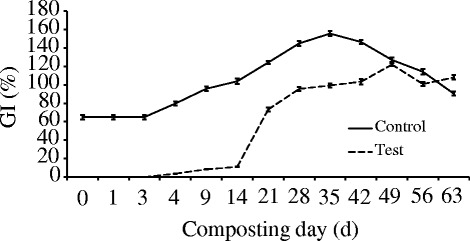
The GI profile during composting

The potential agronomic value of the end product of the compost and its suitability for plant crops must be considered for management of the composting process by evaluating its degree of maturity [1]. Seed germination test is one of the biological methods used to evaluate the maturity of compost. Germination index (GI) was determined in both piles (Fig. 6). The control pile gave a GI of 65.05 % at the initial stage of composting, which exceeded the threshold limit of 60 % stated by Zucconi and de Bertoldi [42] to reduce the phytotoxicity to levels consistent with a safe soil application. For the test pile, the GI was 0 % in the initial days. Then, after the 14 days of composting, there was a significant increase in the GI and it exceeded the threshold limit from 21 days. The GI reached its highest value during the cooling phase for both piles, although significant increases occurred from the thermophilic phase; at the end of composting, this index underwent a slight depression. El Fels et al. [32] reported a similar result by composting two species mixtures (co-composting of sewage sludge-lignocelullosic waste).

At the first stage of composting, the values of the GI are often low because of the inhibitory effect of excessive NH_4_^+^ on the seed germination, but the GI always follows an increasing trend with the proceeding of composting [43]. The result of our current and previous study showed a similar trend [10]. The high value of the GI from the thermophilic phase can be explained by a great reduction of phytotoxic substances. During the cooling phase, the GI continues to increase and generally obtains its highest value with the occurrence of stable organic matter, and the enrichment of humic substances and nutrients [42]. The fall of the GI at the end of composting can be partly explained by the action of phytotoxic substances, and by the high ionic charge of the water-soluble extracts and their electrical conductivity which may cause osmotic effects. This effect is caused particularly by the concentration of mineral elements such as Cu and Zn, which showed an inhibitory effect on germination [32, 44].

### Microbiological analysis

#### E. coli

**Fig. 7:**
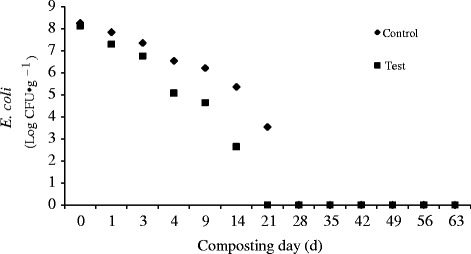
The *E. coli* profile during composting

Our previous works showed that the *E. coli* and other pathogens could be entirely killed with the addition of CaCN_2_ in the cow manure composting [10, 45]. And all of the tested *E. coli* strains with different types of serogroups were quickly inactivated in the compost piles added CaCN_2_ [10]. The present study further confirmed such effect during a forced-aeration static-pile system in which maize straw was a bulking agent. As shown in Fig. 7, the *E. coli* was not thoroughly detected from 21 days in the test pile when from 28 days in the control pile. The addition of CaCN_2_ shortened the time to inactivate *E. coli* in the compost pile.

### Mesophilic and thermophilic bacteria

**Fig. 8:**
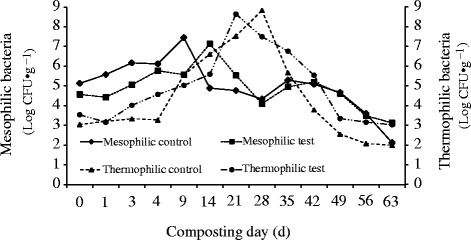
The mesophilic and thermophilic bacteria profile during composting

At the first stage of composting, mesophilic microbial community converts easily degradable substrates and this activity increases temperature [46]. As temperature rise and exceed the tolerance limit of mesophilic microorganisms, thermophilic microorganisms dominate the microbial community [47, 48]. In this study, the development trend of the mesophilic and thermophilic bacteria was similar in both piles (Fig. 8) and was in agreement with the works of the above authors. With the addition of CaCN_2_, the initial population of mesophilic and thermophilic bacteria fluctuated slightly, but not significantly. At the end of composting, the population decreased by 58.67 and 31.51 % for mesophilic bacteria in the control pile and 34.43 and 14.41 % for thermophilic bacteria in the test pile. The decreasing rate of mesophilic bacteria was greater than thermophilic bacteria, which was related to the long thermophilic phase. The addition of CaCN_2_ did not significantly influence the total average population of mesophilic bacteria while increased that of thermophilic bacteria. It was because the longer and continuous thermophilic phase occurred in the test pile and the temperature was also much higher than the control pile (Fig. 8).

## Conclusions

The work presented in this paper has demonstrated the feasibility of applying CaCN_2_ during minimally composting of manure with maize straw in a forced-aeration static-pile system. This provides a potential treatment process to improve the overall economic and environmental performance of manure recycling. The results of the investigation are summarised as follows.

- The time to reach the high temperatures was delayed but the maximum temperature increased and the duration of the thermophilic phase was lengthened with the addition of CaCN_2_.
- The environment suitable for composting process was not influenced by the addition of CaCN_2_.
- The addition of CaCN_2_ increased the percent T-N but decreased C/N ratio during composting.
- With the addition of CaCN_2_, the phytotoxicity of the composting was decreased.
- The time to entirely inactivate *E. coli* in the composting could be shortened through adding 2 % CaCN_2_ into the compostable substrate.

## Abbreviations

CaCN_2_: Calcium cyanamide
EMB: Eosin-methylene blue agar
GI: Germination index
MB: Mesophilic bacteria
PVC: Perforated polyvinylchloride
TB: Thermophilic bacteria
T-C: Total carbon
T-N: Total nitrogen
T-P: Total phosphorus
